# Extending Wireless Rechargeable Sensor Network Life without Full Knowledge

**DOI:** 10.3390/s17071642

**Published:** 2017-07-17

**Authors:** Najeeb W. Najeeb, Carrick Detweiler

**Affiliations:** Computer Science and Engineering Department, University of Nebraska, Lincoln, NE 68588, USA; carrick@cse.unl.edu

**Keywords:** charging algorithm, no knowledge charging, wireless recharging sensor network, wireless power transfer, unmanned aerial vehicle

## Abstract

When extending the life of Wireless Rechargeable Sensor Networks (WRSN), one challenge is charging networks as they grow larger. Overcoming this limitation will render a WRSN more practical and highly adaptable to growth in the real world. Most charging algorithms require a priori full knowledge of sensor nodes’ power levels in order to determine the nodes that require charging. In this work, we present a probabilistic algorithm that extends the life of scalable WRSN without a priori power knowledge and without full network exploration. We develop a probability bound on the power level of the sensor nodes and utilize this bound to make decisions while exploring a WRSN. We verify the algorithm by simulating a wireless power transfer unmanned aerial vehicle, and charging a WRSN to extend its life. Our results show that, without knowledge, our proposed algorithm extends the life of a WRSN on average 90% of what an optimal full knowledge algorithm can achieve. This means that the charging robot does not need to explore the whole network, which enables the scaling of WRSN. We analyze the impact of network parameters on our algorithm and show that it is insensitive to a large range of parameter values.

## 1. Introduction

With the increase in popularity of Wireless Sensor Networks (WSN), the need for sustainable networks becomes more and more prominent. Low cost sensors may make it appealing to replace sensor nodes when their power level is depleted. Simply replacing sensor nodes is not only costly but also harmful to the environment [1]. In addition, finding the sensor nodes that need replacement or charging is challenging [2].

There are many approaches to increasing the life of a WSN ranging from improving communication protocols [3], smarter energy management [4], energy harvesting [5], reclamation and replacement [6], to periodic recharging [7]. In this work, we focus on using an Unmanned Aerial Vehicle (UAV) [8], to charge a WRSN. This has benefits such as the ability to reach locations that are harder to access, but also adds other challenges such as higher energy use while in flight. Among works that recharge a WRSN with a Mobile Charging Robot (MCR) [7], many aim to address the optimal path planning problem [9] and the charge selection problem simultaneously [10,11]. This renders the charging problem hard, nondeterministic polynomial complete (NPC) [12], causing the solution to be infeasible for large-scale WRSN. Even when utilizing several MCR units, the problem is still shown to be nondeterministic polynomial hard (NP-Hard)  [13].

Our work decouples the charging problem from path planning, resulting in a computationally tractable solution. Our charging solution also requires no a priori knowledge of the current nodes’ power levels, enabling it to scale with large WRSN. Reducing data collection in WRSN not only reduces the transmission power consumption at each sensor node, but also the overall computational cost across the network. Reducing the data flow requirements on a WRSN enables it to scale better.

Large-scale WSNs are defined as either networks covering large areas or networks of high density [14]. While high density with small areas can still be dealt with by charging multiple nodes at the same time [9], collecting power information means more overall node power is wasted delegating power information. As for large area WSNs, they may reach a size that is impossible for an MCR to fully cover. While advancement in Lithium-Ion batteries [15] may enable an MCR to overcome parts of this limitation, the need for the network to provide power information becomes more expensive with larger areas. Another solution is to reduce the cost of information collection by simultaneously collecting information while charging [16], and providing an optimal policy for power allocation. Eliminating the need for power information delivery reduces the load on the WRSN. We propose ANLPP (All Network Least Possible Probability), an algorithm that does not require a priori knowledge of WRSN power levels. To the best of our knowledge, this is the first non-exhaustive attempt to charge a WRSN with no a priori knowledge of the sensor nodes’ power levels.

**Figure 1:**
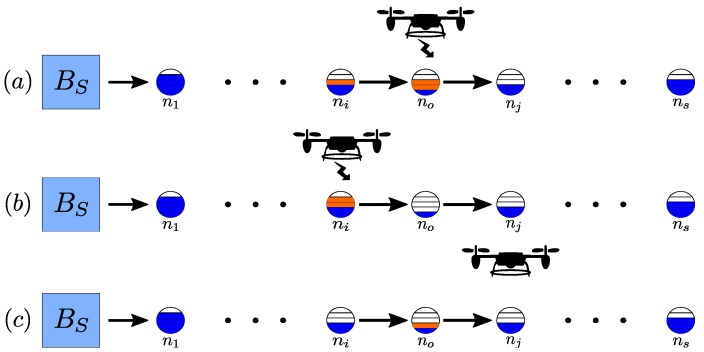
An unmanned aerial vehicle (UAV) charging a wireless rechargeable sensor network (WRSN) based on the stopping point: (**a**) optimal stopping point, (**b**) early stopping, (**c**) late stopping. Blue indicates power level of sensor node, orange added power by wireless charging.

We consider without the loss of generality a line graph network topology shown in Figure 1, similar to [10]. Assuming path planning is already provided then any network topology can be reduced to a line topology, in the sense of its path plan. The line topology is also appropriate since we use a UAV as the MCR [17], which is able to quickly traverse the area. This layout is also very practical and used to cover, operate, or maintain line based systems such as power lines [18], oil lines, bridges [19], rail-roads [20], and border protection. Line layout is also applied when the robots are constrained to a single path of movement in a rough terrain, or when they are moving along a rail [21]. We consider the definition of WRSN life as the time a percentage of the network dies [22,23].

The general problem that we consider in this paper is the stopping point in an exploration path, and the amount of power to charge each node. The problem is as follows: given a set of sensor nodes on a line graph as demonstrated in Figure 1, with each node at an unknown power level, and a UAV as an MCR with limited power, increase the life of the WRSN as close to optimal as possible. The network dies once *k* nodes reach zero residual power. There is no knowledge of the exact power level of any node prior to the UAV visiting the node, making it only possible to identify the nodes that need to be charged after they are visited. On the other hand, we assume the nodes have a known discharge rate and a known power level at a previous time instance. The network size is such that if the UAV visits the whole network, it will only be able to charge a single node. The UAV can charge one node at a time, but may still charge several nodes in a single round-trip. It needs to identify the furthest beneficiary point of exploration that helps in determining the best set of nodes to charge. Charging this set extends the network life close to what an optimal algorithm with full knowledge can achieve.

One of the key challenges associated with charging WRSN is determining which node to charge if not all nodes can be charged. Figure 1 shows a WRSN with a UAV used as the MCR and the challenges associated with charge selection. This figure shows three stopping points for the charging algorithm, the best stopping point Figure 1a, an early stopping point Figure 1b, and a late stopping point Figure 1c. A full knowledge algorithm can determine the optimal stopping point for a UAV; in Figure 1, it is sensor node $n_{o}$. Reaching this point enables the UAV to charge the set of nodes that will extend the network life to the most, while still enabling the UAV to return back to $B_{S}$. As can be seen in Figure 1a, the UAV was able to bring the two visible nodes to greater than 50% of their power capacity. While stopping short at node $n_{i}$, as shown in Figure 1b, will enable charging node $n_{i}$ to a higher level, and prevent the UAV from charge node $n_{o}$, this, in turn, results in an early death of the WRSN. On the other hand, reaching node $n_{j}$, Figure 1c, will consume more power, causing the UAV to not have enough power left to charge $n_{i}$ or charge the nodes to less levels, leading to a lower network life extension. Without prior knowledge of the sensor nodes’ power levels, determining this stopping point becomes challenging. The need to identify the low power nodes and the stopping point is crucial for optimal charging of a WRSN. This necessitates the need to identify the lowest power nodes in the network fast, and at a low cost. Once a charging algorithm can predict, with some certainty, that it has identified the nodes with lowest power levels, it may terminate further exploration.

We use a probabilistic approach to address the charging of large scale WRSN. Our algorithm utilizes probability to make stopping decisions. We also use a charging algorithm that charges a subset of nodes only considering power levels observed during flight. Determining the power level of nodes in a WRSN is very important to optimally charge the network. Charging and exploring a WRSN based on the actual collected power information, at the time of exploration, enables the conservation of power and scalability of efficient WRSN charging.

The main contributions of this paper are:

- Presenting ANLPP, a novel algorithm that increases the life of a WRSN with no knowledge of sensor nodes’ power levels and without the need to fully explore the network. Simulation results showing that ANLPP performs on average at 90% of what an optimal full knowledge algorithm could achieve.
- Analysis of WRSN parameters that impact the performance of ANLPP, and show that ANLPP is tolerant to a wide range of WRSN parameters.

The rest of this paper is organized as follows. Section 2 describes related work. Section 3 presents the formal definition of our problem. Section 4 defines the power model of our WRSN. In Section 5, we show the exact probability of a node to be at a certain power level, and in Section 6 we then derive the upper bound on the sensor power probability. Section 7 describes the general layout of the algorithm that addresses our problem, and, in Section 8, we describe several approaches to address the stopping sub-problem. Section 9 shows the full knowledge optimal solution our algorithm is compared against. In Section 10, we present our results and discuss them. We conclude with Section 11 presenting our conclusions with an outlook on future work.

## 2. Related Work

Previous efforts related to wireless rechargeable sensor networks can be divided into several categories. Some address the power replenishment using different techniques; other work addresses different network types. While some authors address scalability of WRSN, others address the levels of power knowledge needed by the charging algorithm. We now discuss these efforts and how they relate to our work.

### 2.1. WRSN Power Replenishment

There are a variety of methods to replenish the power level of sensor nodes in WRSN. For instance, energy harvesting is adopted to recharge the sensors in WRSN using solar cells [24]. Other sources like wind [25], vibration [26], difference in temperature [27], and even human blood pressure are also used for energy harvesting [28]. However energy harvesting is unpredictable and hard to control [29] because the ambient source of power is unknown or unstable. On the other hand, a more predictable replenishing technique involves wireless power transfer from a battery based power source. Wireless charging can be implemented in several methods. One way to charge nodes is to use radio frequency [30]. It has been shown that, using a radio frequency (RF) harvester [31], both ambient RF power from different sources or from dedicated transmitters can be utilized  [32]. Another method is using strong-coupled magnetic resonance [33]. In our work, we adopt the strong-coupled magnetic resonance technique due to the power hazards and limitations [34], and the rapid decay of power transfer efficiency with increased distance [35], in radio frequency charging.

Mobile sensor based networks can also benefit from wireless power transfer. Using stationary charging stations with partial coverage, the WRSN life can be extended [36]. One or more charging stations can be used to extend the life of a mobile WRSN [37]. In a stationary sensor based WRSN, a single MCR [38] may be deployed to periodically travel inside the network and charge sensor nodes or several MCR units can be used [39]. In our work, we focus on a single MCR to charge a stationary sensor based WRSN. We also do not require exhaustively visiting the whole network.

### 2.2. WRSN Scalability

We focus on WRSN scalability as a network expanding over a large area , as defined in [14]. An optimal solution using linear programming was proposed, after analysing the optimal movement strategies of the MCR [40]. However, solutions requiring high overhead or simply based on sensor node location are impractical for large scale WRSN [41]. Scalability in WRSN has been addressed using several MCR units for charging [39], by defining their coordination and recharging activities. The use of several MCR units led to new questions that needed to be addressed. Identifying the minimum number of MCR units needed to maintain a consistent and efficient charging mechanism was proved to be an NP-Hard problem [42] by reducing it to the Distance Constrained Vehicle Routing Problem. An approximation algorithm was proposed as a solution, where a linear constraint is removed converting the problem to a relaxed version. In another approach [10], an MCR was used to charge another MCR to enable scalability of WRSN charging. While it is possible to show that several nodes can be charged at the same time [9], it only addresses charging high density large-scale WRSN. We provide a solution for large area large-scale WRSN using a single MCR.

With the increase in WRSN scale, the cost of data propagation significantly increases. In an effort to reduce sensors’ energy on reporting data to the sink, studies have been conducted to investigate efficient algorithms to both replenish energy levels of sensors and collect data from them simultaneously [43], or in two stages [44]. Efficient algorithms have been developed to schedule minimum mobile devices for energy replenishment and data collection in a WRSN [45], such that the network life can be prolonged with no limits. Showing this problem is NPC [45]. Combining energy replenishment and data collection with multiple sinks was also shown to be an NP-Hard problem [13], requiring full knowledge of sensors’ power levels. A battery-aware mobile energy replenishment and data collection method is proposed in [46] to visit locations such that sensors within the MCR range are charged, while decisions are made according to real-time energy information obtained when visiting head nodes, moving to areas with lower power levels. This approach requires partial knowledge gained while randomly exploring the WRSN. Our work uses a single MCR that only performs charging with no data collection. On the other hand, our work reduces the amount of data propagation, by requiring no power knowledge to be propagated for the charging algorithm.

As WRSNs scale, the cost of collecting energy information becomes impractical. An efficient energy monitoring protocol was designed to address this problem [39]; however, it assumes the use of several MCR units and each MCR requires full knowledge of the nodes and communication protocols. A high level of integration between the charging system and the actual WRSN operation protocols may become problematic with the scaling of WRSN. Most charging solutions require full knowledge of all sensor nodes’ power levels. Full knowledge is also required when addressing the charging problem as an optimization problem [7], where the ratio between the MCR vacation time over cycle time is maximized. While sensor nodes are less energy demanding, most of the energy is consumed by the transceivers [47], and more power can be saved by reducing energy information delegation. This need for energy information in turn limits the scalability of WRSN. We attempt to address WRSN scalability by eliminating the need for a priori energy information.

### 2.3. Power Knowledge

Most of the solutions for WRSN require full knowledge of power levels. In a partial knowledge adaptive approach, power level information from representatives of a subset of nodes was used to increase the life of a WRSN [48]. They deploy a single MCR that adapts its trajectory based on the energy dissipation rate of the selected representatives, assuming they reflect the behaviour of nodes in their locality. While this approach requires less information, it still requires a priori knowledge.

Zero knowledge based algorithms using several MCR units were presented for charging WRSN [49]. The authors compared several charging algorithms, two of which were zero knowledge algorithms: centralized charging (CC) and distributed charging (DC). In both DC and CC, the MCR exhaustively visits all the nodes in its designated or negotiated region, respectively. They reported that the no knowledge algorithms had lower performance when compared to other full or partial knowledge based approaches when considering network life. In this work, we present both a no-knowledge and a full-knowledge based approach using a single MCR. We did not compare our work to other zero-based knowledge approaches, since we use a single MCR while others use several. We also did not compare our work to other single MCR algorithms, since they require full sensor nodes’ power knowledge. A full-knowledge approach assumes a priori knowledge of all sensor nodes’ power levels, while a no-knowledge approach requires only the discharge rates and power levels at the last time of network traversal. Our no-knowledge approach performs close to the full-knowledge algorithm.

## 3. Problem Formulation

In this section, we give the formal definition of our general problem. Suppose that there is a base station $B_{S}$ and a set of nodes of size *s*

$$N=\{n_{1},n_{2},\cdots \;n_{s}\},$$

where the nodes are independent, and each node $n_{i}$ has a residual energy level $e_{i}$. Without the loss of generality, assume all nodes have the same battery capacity $E_{S}$, and the set $N_{e}$ represents the set of residual energy levels of all nodes

$$N_{e}=\left\{e_{1},e_{2},\cdots \;e_{s}\right\}.$$

The set $N_{d}$ represents the distance from node $n_{i-1}$ to node $n_{i}$, and each node is placed at distance $d_{i}$ from the previous node, the first node is inserted at distance $d_{1}$ from the base station $B_{S}$, and so on

$$N_{d}=\left\{d_{1},d_{2},\cdots \;d_{s}\right\}.$$

A single MCR with total power $E_{c}$ is utilized to charge a subset of the WRSN. We define the power needed to align the MCR charging coils to the center of the sensor node’s receiving coils, referred to as concentrically localize the MCR and a node, λ, the target power level of each node to be charged as *l*, total localization and charge power as *C*, total number of sensor nodes to charge as *u*

$$C=u\times \lambda +\sum_{i=0}^{u}l-e_{i}.$$

We define Υ as the maximum feasible subset of nodes the MCR may charge to achieve the highest possible life extension, and Γ is the point enabling the MCR to reach the furthest node in Υ

u=|Υ|,

$$\Upsilon =\{n_{t1},...,n_{tu}\}\subset N,$$

$$\Gamma =\sum_{i=1}^{tu}d_{i}.$$

Charging Υ leads to the maximum WRSN life extension Ψ. We define *M* as the total moving power consumed by the MCR, and this includes the power needed to reach the furthest node to visit and return to $B_{S}$. Assuming the unit moving cost is $\Lambda_{m}$

$$M=\Lambda_{m}\times \Gamma ,$$

and the power constraint formula is such that

$$E_{c}\geq C+M.$$

Each sensor node discharges power at either rate *r* or zero, based on a discharge function *g* and a discharge probability *p*, we use a fixed discharge probability for each run similar to [46]

$$g\left(n\right)=\left\{\begin{array}{cc} r, & \mathrm{if}\;\mathrm{node}\;\mathrm{is}\;\mathrm{activated},\\ 0, & \mathrm{otherwise}. \end{array}\right.$$

Finally, we define the network life as the time until *k* nodes reach zero residual energy:

$$k=\sum_{i=1}^{s}f(n_{i}),$$

$$f\left(n_{i}\right)=\left\{\begin{array}{cc} 1, & \mathrm{if}\;e_{i}\;\mathrm{equals}\;0,\\ 0, & \mathrm{otherwise}. \end{array}\right.$$

The objective is to maximize the increase in network life. With no prior knowledge of $N_{e}$, this objective is accomplished by identifying:

- The best point to stop exploring and return to $B_{S}$, γ,
- The best possible set of nodes to charge, υ.

Stopping at point γ leads to identifying υ. Charging υ results in a network life increase ψ. The problem is to bring ψ as close to Ψ using a least intrusive no knowledge charging algorithm.

## 4. WRSN Power Model

In this section, we define the parameters involved in modelling our sensor nodes from the power perspective. Considering the discharge function g(n), where nodes are asleep (inactive) or awake (active) and consuming power *r*, the nodes’ power level are always decreasing. We also assume node discharge independently of each other and randomly, i.e., random events trigger the discharge. Define *T* as the time interval elapsed since node was at power *W*.

Other properties and definitions of sensor nodes are:

- Sensors have the same maximum power capacity $E_{S}$,
- Without loss of generality, we can assume that all nodes start at the same power level. The last known power level of a sensor node is *W*.
- The probability of a sensor node discharging at each time unit is *p*.
- Without loss of generality, we can assume that all nodes have the same discharge rate *r*.
- The power level of the currently explored sensor node is *Y*.

Given the sensor node model and the discrete nature of the discharge function, we can compute the probability of a node to exist at each power level. We start by computing the exact probability of a certain power level, and later compute the expected power level of a node and identify probability bounds. Table 1 lists the notations used throughout this work.

## 5. Exact Probability of a Node’s Existence at a Certain Power Level

In this section, we compute the exact probability of a single node to be at a certain power level, by answering the following question: given a node with power level *Y*, what is the probability of such a node, or one with less power, to exist in the network? We assume the following:

- *T* time units have elapsed,
- the node was at power level *W* before *T* time units,
- discharge probability is *p* at each time unit.

We start by identifying how many discrete steps *t* are needed for a node to reach power level *Y*. Due to the discrete nature of discharge and knowing the discharge rate *r*, we can define *t* as

t=(W−Y)/r.

Our problem is now simplified to finding the probability of *t* or more discharges to occur in *T* steps. We treat this as Bernoulli trials with success= *p* and failure= 1−p. The probability of exactly *t* discharges to occur will be

Ttpt(1−p)(T−l),

and the probability of a node with power less than or equal to *Y* will be

ξ=∑j=tTTjpj(1−p)(T−j).

While the above formula can be used to find the total probability, we face two main problems when dealing with it. First, the numbers become significantly small with the increase of *j* (proportional to time elapsed). When using the parameters needed to simulate a large scale WRSN, the precision exceeds our computer’s computational precision. Each computed probability component becomes zero. Even with an increase in computational power, we still face another problem. This calculated probabilistic value does not give us an exact prediction, increasing the chances of making mistakes if these values are used. Due to these limitations, we need to find a better and more scalable solution. We turn to utilizing an upper bound on our desired probability estimations instead of computing the exact probability. An upper bound will be more helpful in identifying highest possible values without having to consider exact probability and variance.

## 6. Probabilistic Bound on Node Existence

We develop an upper probabilistic bound on the sensor node’s power level based on the node model described in Section 4. Given the problem formulation from Section 4, and the probability discharge function *P*, the expected charge level of each node is defined as E(X):

$$E\left(X\right)=W-\sum_{t=1}^{T}P\left(t\right)g\left(t\right)=W-\sum_{t=1}^{T}\left(p\times r+\left(1-p\right)\times 0\right),$$

(1) $$\begin{array}{c} E(X)=W-prT. \end{array}$$

For unbiased values of *p*, E(X)=W is the highest possible value, while highly improbable. The lowest possible value E(X)=W−rT is also highly improbable.

Our interest is to be able to identify the probability of a node being at or below a certain power level, *Y*. Several tail probability estimation methods exists [50]. While Markov inequality can easily be applied, it provides a relatively loose bound. Other tighter bounds may also be applied, like Chebyshev Bounds, or Chernoff Bound known as the tightest bound. Since the nodes are independent and the discharge at each step is both random and independent, the Chernoff Bound [51,52] conditions are satisfied, and the bound may be applied to give an upper bound to this probability. We use the multiplicative form of the Chernoff Bound:

(2) $$\begin{array}{c} P_{r}\left[X\leq \left(1-\varepsilon \right)E\left(X\right)\right]\leq e^{-\frac{\varepsilon^{2}}{2}E\left(X\right)}. \end{array}$$

Let us assume we wish to identify the probability of a node being at power level *Y* or less, where *Y* is always less than E(X). This is equivalent to setting

$$\varepsilon =\frac{E(X)-Y}{E(X)}.$$

The probability becomes

(3) $$\begin{array}{c} P_{r}\left[X\leq Y\right]\leq e^{\frac{{\left(W-prT-Y\right)}^{2}}{2(prT-W)}}. \end{array}$$

This probability is a function of four parameters, the probability *p* of a node discharging at any time instance, the average discharge rate *r*, the time elapsed *T* since the moment the sensor node power level was *W*, and the node maximum power level *W*.

Equation (3) gives us an upper bound on the probability of finding a certain node at power level *Y* or less. Section 10.1 shows how close our simulations are to this bound. This bound can be utilized to assist an algorithm in identifying nodes at low power levels with high certainty.

## 7. Exploration and Charging Algorithm

In this section, we discuss our algorithm, with three stopping methods. Our objective is to identify, without full knowledge of network power levels, the shortest distance the MCR should travel, γ, and the set of nodes, υ, to charge that increases the life of a WRSN to, ψ, near optimal increase. We start by describing our algorithm, and then show how the stopping decision can be made using three progressive methods: Naive approach, Minimum Only approach, and finally, our ANLPP approach. We then analyze the performance of the algorithm.

Our Explore algorithm (Algorithm 1) successively explores the WRSN nodes sequentially, and performs three steps after visiting each new sensor node in the network:

1. determine the subset of visited nodes to charge υ,
2. decide whether to move forward and visit more nodes or terminate exploration, define γ,
3. when no more nodes are to be visited, return home and charge nodes in υ on the way back.

**Algorithm 1** Find exploration termination point.**Require:** MCR▹ The traversing UAV**Require:** *N*▹ List of network nodes 1: **procedure** Explore(*N*) 2:     vNodes←empty▹ List of visited nodes 3:     υ←empty▹ List of nodes to charge 4:     C←zero▹ Charge power 5:     **for**
i←1...sizeofN
**do** 6:         power← Get MCR Power Level 7:         hp← calcualte power needed to return home 8:         υ,C,l←charge(vNodes,power−hp)▹ Get the charging list, power needed to charge, and new power level 9:         power←power−C−hp▹ Power left, after charging and returning home reserves10:         **if**
canVisitMoreNodes(power,i)
**then**▹ Based on location and power level decide to continue or terminate11:            visit next nodei and read node power12:            add node to vNodes13:         **else**14:            Break15:         **end if**16:     **end for**17:     return to base and charge nodes in υ18: **end procedure**

The Explore algorithm (Algorithm 1) is an online algorithm, which starts when the MCR leaves $B_{S}$ and terminates once the MCR returns to $B_{S}$. It also lets the MCR know when it should go back to $B_{S}$. The algorithm can cope with unpredicted power consumption by the MCR, since it monitors and updates the power availability as new sensor nodes are explored (line 6). The algorithm also reserves power facilitating the return to $B_{S}$ at all times, hp, and updates this value online (line 7). To identify υ, the algorithm invokes a charging algorithm (line 8). If the MCR has enough power left to visit another node, then the MCR is instructed to visit the next node and move forward. If the MCR does not have enough power to charge and return home, then the exploration is terminated, at γ, and the MCR returns to $B_{S}$ and charges the nodes in υ.

Determining υ is done by implementing a charging algorithm. Our Charge algorithm (Algorithm 2) is similar to the Greedy Plus algorithm from [12], but it differs in the knowledge requirements, discharge behaviour and localization cost. Greedy Plus was also demonstrated on a line topology. In Charge, knowledge builds up as the charging unit traverses the network. The Charge algorithm assumes a fast moving MCR, leading to a round trip from $B_{S}$ back to $B_{S}$ in less time than what is needed between two sensor node discharges. The Charge algorithm will sort the nodes based on their power level (line 2), and adds the low power nodes to the charging list sequentially (lines 13–15), until the MCR has no more power to bring the list of nodes proposed to be charged, $\upsilon^{\prime }$, to the next power level (line 17).

The time complexity of Algorithm 1 is $O(n^{2}log\;n)$. The execution of lines 1–4 is constant time O(1). The For loop in line 5 renders an O(n) execution time of everything inside the loop. Line 6, retrieving the MCU power level, is O(1). Line 7, computing the power needed to return home, is O(1) assuming the data is stored in a lookup table. The complexity of the Charge algorithm is O(nlogn), analysis given in the next paragraph. The execution time of all implementations of canVisitMoreNodes algorithm is O(1), and this will be analyzed later with each implementation. All other operations are O(1). This yields in a total execution time of $O\left(1\right)+O\left(n\right)\times \left(O\left(nlog\;n\right)+O\left(1\right)\right)=O\left(n^{2}log\;n\right)$.

The time complexity of Algorithm 2 is O(nlogn). The first operation is a sort, and a quick sort would give O(nlogn). Lines 2–6 are each O(1). The For loop at line 7 renders an O(n) execution time of everything inside the loop. All operations inside the for loop each take O(1). This renders the whole algorithm taking O(nlogn)+(O(n)×O(1))=O(nlogn). The time complexity of all three implementations of canVisitMoreNodes is O(1). Detailed analysis of each one will be presented after each implementation.

**Algorithm 2** Find subset to charge.**Require:** λ▹ Localization cost in terms of power1: **procedure** Charge(vNodes,power)2:     sNodes←sort(vNodes)▹ Sort visited nodes based on power3:     υ←empty▹ List of identified nodes to charge4:     $\upsilon^{\prime }\leftarrow empty$▹ List of candidate nodes to charge5:     C←0▹ Total power used for charging6:     l←0▹ Target power level to charge the nodes in υ7:     **for**
i←1...sizeofsNodes−1
**do**8:         sPower← power level of $i^{th}$ node in sNodes▹ Source node power level to consider9:         tPower← power level of $i^{th}$ + 1 node in sNodes▹ Next possible target power level10:         $\upsilon^{\prime }\leftarrow \upsilon +$$i^{th}$ node in sNodes▹ Add node to candidate list11:         neededPower=λ + ((tPower − sPower) × (1 + sizeof $\upsilon^{\prime }$));▹ Power needed to use new target power level12:         **if**
power≥neededPower
**then**13:            $\upsilon \leftarrow \upsilon^{\prime }$▹ Candidate list is feasible14:            C=C+neededPower▹ Charging power update15:            l=tPower▹ Target power level update16:         **else**17:            **return**
υ,C,l▹ Early termination due to lack of power to charge the whole list18:         **end if**19:     **end for**20:     **return**
υ,C,l21: **end procedure**

Determining the stopping point γ is done by implementing canVisitMoreNodes, where canVisitMoreNodes returns False once γ is reached. The next section addresses possible methods to implement canVisitMoreNodes.

## 8. Stopping Methods

Our implementation of the movement or termination decision function canVisitMoreNodes of Algorithm 1 (line 10) is based on the amount of power left on the MCR. The power left is the power available after subtracting both the power needed to charge, *C*, and the power needed to return to $B_{S}$, hp. While other parameters could be considered for canVisitMoreNodes, we show how power is the main factor. This movement decision-making is what we will describe in more detail based on different parameters. The canVisitMoreNodes procedure implementation can be done in several ways, and, in the next subsections, we will address our three methods addressing making this decision.

### 8.1. Naive Approach

Our first implementation of canVisitMoreNodes (Algorithm 3) is based only on travel power cost. That is, the algorithm will calculate how much power is needed to visit the next node, $n_{i}$, as ($d_{i}\cdot \Lambda_{m}$), which is the distance to reach node $n_{i}$ times the power needed to move that distance, and check if the power left is enough to complete that trip (line 2). If there is enough power left, it returns True, indicating to keep exploring further; otherwise, it returns False, giving an indication to stop exploration. The time complexity of Naive (Algorithm 3) is O(1). It is clear that each operation is an arithmetic operation or comparison taking constant time, O(1), in the algorithm.

**Algorithm 3** Naive approach for canVisitMoreNodes.**Require:** $N_{d}$▹ Set of distances between nodes $=\{d_{1},d_{2},...,d_{s}\}$**Require:** $\Lambda_{m}$▹ MCR moving power consumption per move unit1: **procedure**
canVisitMoreNodes(power,i)▹ power: amount of power remaining, i: index of next unvisited node2:     **if**
$power\geq 2\cdot (d_{i}\cdot \Lambda_{m}$) **then**▹ Multiply by 2 to accommodate for returning3:         **return**
True4:     **else**5:         **return**
False6:     **end if**7: **end procedure**

The Naive approach is good at preventing the MCR from getting stranded. The Naive approach performs satisfactorily when the low-power sensor nodes in *N* are located close to $B_{S}$ because there will be a high chance of including the lower power nodes in υ. However, the algorithm naively assumes it will be able to find the low power nodes before it runs out of power. The combination of Charge and Naive approach attempts to charge all the nodes it has visited so far, and only explore with the remaining power. This prevents the discovery of nodes with lower power that are further down the network. The network life extension is minimal or even inexistent if the charged nodes are part of the *k* nodes to drop. Naive fails to extend the life of the network if the low power nodes are not close to $B_{S}$. The next approach attempts to address having low power nodes further down the network.

### 8.2. Minimum Only Approach

Algorithm 4 shows the Minimum Only implementation of the procedure canVisitMoreNodes. The Naive approach attempts to charge almost all the nodes it encounters until it runs out of power. Intuitively sacrificing a node from υ in exchange for more exploration may lead to finding more lower powered nodes. The Minimum Only approach builds on this intuition and sacrifices high power nodes in υ, until the nodes with the lowest power are identified as the ones that need charging in υ. The algorithm uses the probabilistic bound Equations (2) and (3) to calculate the probability, pLo, of a node existing at power level less than or equal to the lowest power level found in υ. The algorithm uses pLo to determine if further exploration should be conducted or terminated.

**Algorithm 4** Minimum Only approach for canVisitMoreNodes.**Require:** *N*▹ Set of network sensor nodes**Require:** λ▹ Localization cost in terms of power**Require:** $N_{d}$▹ Set of distances between nodes $=\{d_{1},d_{2},...,d_{s}\}$**Require:** minThreshold▹ Minimum probability threshold, tuning parameter1: **procedure**
canVisitMoreNodes(power, υ, *C*, *l*, vNodes, *i*)▹ l: node target power level2:     **if**
$power\geq (d_{i}\cdot \Lambda_{m}$) **then**3:         **return**
True4:     **else**5:         minNeededPower←λ+l−min(υ)▹ Power needed to charge lowest power node in υ6:         **if**
C>minNeededPower **then**▹ Proceed if charging power is greater than power needed to charge lowest node7:            pLo←findProbLess(min(υ))▹ Probability of lower power nodes to exist, less than the lowest in υ8:            **if**
pLo>minThreshold
**then**9:                **return**
True10:            **else**11:                **return**
False12:            **end if**13:         **end if**14:     **end if**15: **end procedure**

The time complexity of Minimum Only (Algorithm 1) is O(1). It can be seen that almost all operations are of constant time O(1). There are two method calls finding the minimum (line 5) min(υ) and probability computation findProbLess that need to be analyzed, given that finding the minimum node min(υ) (line 5) in the charging list υ is O(1). This is possible because the charging list is a sorted list, so accessing the minimum element takes constant time. In addition, computing the probability based on Equation (3) (findProbLess) is a constant time operation O(1). All this renders the Minimum Only approach O(1).

The algorithm also defines a minimum probability, minThreshold, that must be exceeded, which enables the algorithm to keep exploring until it finds low power nodes. The algorithm also guarantees that it charges at least one node. This is achieved by making sure the total power needed to charge, *C*, is greater than the power needed to charge at least the lowest power node in υ identified as minNeededPower (lines 5–6). If the power used to charge is only used to charge a single node, then we terminate the exploration, since any movement further may not increase the life of the network.

Naive was not able to discover low power nodes located further down the network. While Minimum Only manages to overcome this limitation, and charge low power nodes located far from $B_{S}$. On the other hand, there is a chance it is wasting power. The algorithm makes sure the lowest power node in υ is a low power node. However, there still could be nodes in υ with relatively high power levels, greater than the expected power level, rendering charging them less useful in extending the network life. The existence of high power nodes in υ means either power is wasted in charging high power nodes, or power is wasted charging low power nodes to high levels preventing exploration. The next approach attempts to remove high power nodes from υ.

### 8.3. ANLPP Approach

All Nodes Least Possible Probability approach (ANLPP), our final implementation of canVisitMoreNodes, illustrated in Algorithm 5 builds on Algorithm 4 and minimizes the chances of wasted power. To minimize these chances, we need to minimize the number of high power nodes in υ. This can be achieved by identifying the probability of all the nodes in υ to be of low power. If we utilize our bound for both the highest and lowest power nodes in υ, we can determine if all nodes in υ are within a set of lower power nodes in the network.

**Algorithm 5** ANLPP approach for canVisitMoreNodes.**Require:** *N*▹ Set of network sensor nodes**Require:** λ▹ Localization cost in terms of power**Require:** $N_{d}$▹ Set of distances between nodes $=\{d_{1},d_{2},...,d_{s}\}$**Require:** minThreshold▹ Minimum node probability threshold, tuning parameter**Require:** maxThreshold▹ Maximum node probability threshold, tuning parameter1: **procedure**
canVisitMoreNodes(power, υ, *C*, *l*, vNodes, *i*)2:     **if**
$power\geq (d_{i}\cdot \Lambda_{m}$) **then**3:         **return**
True4:     **else**5:         minNeededPower←λ+l−min(υ)6:         **if**
C>minNeededPower**then**▹ Proceed if charging power is greater than power needed to charge lowest node7:            pLo←findProbLess(min(υ))▹ Probability of lower power nodes to exist, less than the lowest in υ8:            **if**
pLo>minThreshold
**then**9:                **return**
True10:            **else**11:                pHi←findProbLess(max(υ))▹ Probability of lower power nodes to exist, less than the highest in υ12:                **if**
pHi>maxThreshold
**then**13:                    **return**
True14:                **else**15:                    **return**
False16:                **end if**17:            **end if**18:         **else**19:            **return**
False20:         **end if**21:     **end if**22: **end procedure**

ANLPP implementation of canVisitMoreNodes (Algorithm 5) extends the MCR exploration limit under three conditions:

- sufficient power exists in the MCR to explore further,
- there is a high probability of finding nodes with power level less than the minimum power level node in υ,
- there is a high probability of finding nodes with power level less than the maximum power level node in υ.

The algorithm applies all the steps from the Minimum Only approach (Algorithm 4). The algorithm calculates the probability of finding a node with power less than the highest power node in υ as pHi. If pHi is above the predefined maxThreshold, then keep exploring, since there is a high chance of finding nodes with power less than the maximum power node in υ. This minThreshold may be chosen based on the minimum known MCR coverage, or the minimum reasonable certainty needed by the user. This implementation also terminates its exploration once it realizes it can only charge a single node (line 19). The time complexity of ANLPP is also O(1). Most of the operations are of constant time O(1), and the method used are the same methods used in Minimum Only leading to applying the same analysis. Next, we will show the baseline algorithm to compare against.

## 9. Full Knowledge Optimal Algorithm

In this section, we describe the optimal full knowledge algorithm used as a baseline for comparing the performance of our algorithms. This algorithm requires full knowledge of all the power levels of all the sensor nodes in the WRSN.

Given the full knowledge of the power levels of the WRSN, it is possible to identify the set of lowest power nodes in the network. Charging a node involves three main factors: the cost to localize (concentrically align the MCR with it), the cost to reach it, and finally the cost to bring its power level to the target level. When the charging list is determined, the total cost can be computed, and, then, comparing the available power to the total cost identifies the feasibility of charging. Using the Charging Algorithm 2, the target node is the next node after the set of charged nodes, υ, and the (k−1) nodes that may be dropped. Identifying the target node enables us to identify the network’s new minimum power level, thereby allowing us to estimate the life extension of the WRSN.

We take a top-down approach in our realization of an optimal solution, presented in Algorithm 6. Assume the highest possible life increase, while highly improbable, and gradually reduce the life increase until we hit the highest possible one. We start by considering localization cost; knowing the localization power cost, we can identify the highest possible number of nodes to charge, *u*, ignoring all other factors (line 3). Since it is only after *k* nodes deplete their power that the network dies, we can skip charging k−1 nodes. This makes the target node the $N_{u+k}$ node (lines 4–5). This is highly unlikely to be a target, since we are assuming the use of all available power only for localization. Given this target, we compute the needed charging and moving power (line 6). If the existing power is enough to satisfy all power needed for moving, charging, and localization then we are done finding a target (line 7). Otherwise, revert to the next highest power node as a target. This removes a node from the charging list, the second highest node, reducing total localization cost and charging cost. Repeat the power feasibility test until a solution can be found (lines 7–12). The algorithm execution complexity is $O(n^{2}log\;n)$.

**Algorithm 6** Find Optimal Time Increase.**Require:** $E_{C}$▹ Maximum MCR power**Require:** λ▹ Localization cost in terms of power**Require:** *k*▹ Number of possible nodes to drop**Require:** *N*▹ List of network nodes1: **procedure** FindOptimalTimeIncrease(*N*)2:     $sortedN\leftarrow E_{C}/sort\left(N\right)$▹ Sort the nodes based on power3:     $u\leftarrow E_{C}/\lambda$▹ Number of nodes in charge list4:     targetIdx←u+k▹ Index of target node5:     $targetPower\leftarrow sortedN_{targetIdx}$▹ Highest possible target6:     C,M←ComputeNeededPowers(N,targetPower,u)▹ Power needed to charge and move7:     **while**
$E_{C}<C+M+\left(u\times \lambda \right)\;\mathrm{do}$8:         targetIdx←targetIdx−1▹ If not enough power exists then pick a lower target9:         $targetPower\leftarrow sortedN_{targetIdx}$10:         u←u−1▹ Reduce number of charged nodes by one11:         C,M←ComputeNeededPowers(N,targetPower,u)12:     **end while**13:     $timeIncrease\leftarrow (targetPower-sortedN_{k})/r$▹ Time is how long the extra power can be consumed14:     **return**
timeIncrease15: **end procedure**

To compute the time increase, we simply compare the amount of added power to the $k^{th}$ lowest power node. The time increase will be the time it takes to consume the extra power added to the *k* lowest power sensor nodes. This time increase is the lowest possible time increase. A more accurate value will involve the probability of a sensor node discharge. The lowest possible time increase is sufficient for algorithms’ performance comparison when similarly applied to all algorithms.

The power computing algorithm (Algorithm 7) computes the power needed to charge and reach the nodes in the charging list. Travel cost is the cost to reach the furthest node in the charging list (lines 14 and 15). A cost is assigned to each node, defined as the amount of power needed to bring the node to the target power level and the cost to reach it (in terms of power). Based on the cost, the algorithm identifies the lowest cost nodes to charge, and accumulates the costs (lines 9–13).

**Algorithm 7** Compute Needed Power to Charge and Move Given Nodes.**Require:** *d*▹ Distance between two consecrative nodes**Require:** $\Lambda_{m}$▹ Localization cost in terms of power1: **procedure**
computeNeededPower(N,targetPower,u)2:     **for**
node∈N
**do**3:         **if**
nodepower≥targetPower
**then**4:            $cost_{node}\leftarrow \infty$▹ Eliminate nodes beyond maximum reach5:         **else**6:            $cost_{node}\leftarrow targetPower-nodepower+\left(d_{node}\times \Lambda_{m}\right)$▹ Compute cost to reach and charge a node7:         **end if**8:     **end for**9:     **for**
i←1...u
**do**▹ Charge *u* cheap nodes10:         node←find $i^{th}$ lowest cost node in cost set11:         $C\leftarrow C+targetPower-node_{power}$▹ Accumulate charge cost12:         u←u−113:     **end for**14:     pose← furthest position in the first *u* nodes from cost15:     $M\leftarrow pose\times \Lambda_{m}$▹ Compute moving cost, the cost to reach furthest node to charge16:     **return**
C,M17: **end procedure**

## 10. Simulation Results and Analysis

In this section, we will present our simulation results and analyze them. We show the utility and practicality of our theoretical bound. We analyze the results of running our three algorithmic approaches. While all other algorithms assume some level of knowledge or full-knowledge, our approach assumes zero-knowledge. That is why we chose not to compare our simulation to other simulations and algorithms. We conclude this section with the impact of network parameters and algorithm parameters on the overall performance.

We run our simulations using MATLAB (R2016a 9.0.0.341360 64-bit) on an Intel core processor (Haswell, Lincoln, NE, USA) 2.3 GHz 16 cores machine (two processors, eight cores each), and 16 GB RAM. We set-up the WRSN using real world parameters obtained from our previous work [8], shown in Table 2.

Our simulations show that it takes two minutes to run ANLPP over ten thousand networks of over 200 nodes each. This execution time combined with the low memory requirements of ANLPP, for a single iteration (O(n)), means our work could easily be integrated into an MCR with standard computational capabilities.

Since other algorithms in the literature require full or partial knowledge, we do not compare them to our algorithm and simulation results. On the other hand, we compare our results to a full knowledge optimal implementation for charging the WRSN, which is at least as good as the other approaches would be on these networks.

### 10.1. Upper Bound Analysis

**Figure 2:**
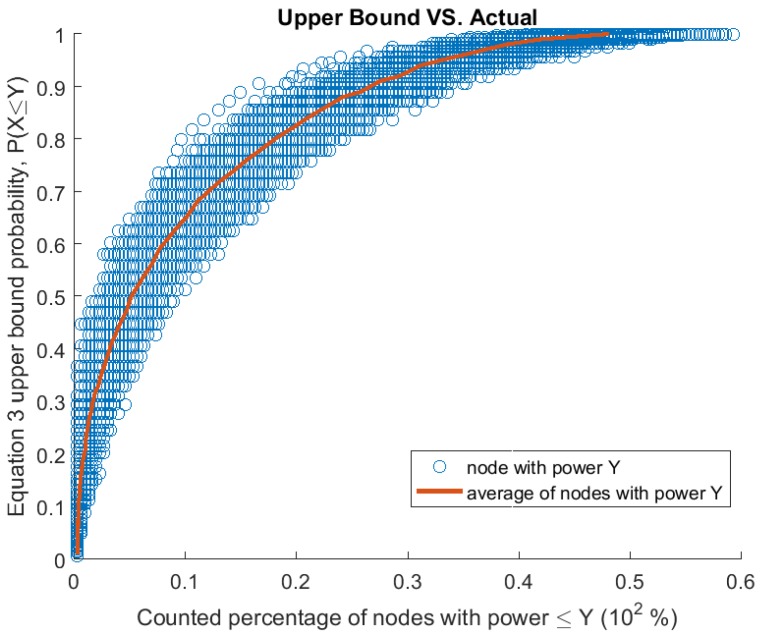
Plotting 10,000 random WRSNs. Each point is a node with residual power *Y*. For each node, the actual percentage of nodes with power ≤Y (*x*-axis) vs. the computed bound (*y*-axis) are plotted.

We start by showing how close our simulation results are to the theoretical bound obtained by applying Equation (3). Figure 2 shows the actual percentage of counted nodes with power less than or equal to a given power level against the calculated theoretical upper bound for the same power level.

Each point in Figure 2 represents counted and computed probability for a power level to exist. We generated 1000 random networks consisting of 300 nodes each. For each node in the network, we identify its residual power level *Y* and perform two tasks:

- We count the number of nodes with residual power less than or equal to *Y*, in the same network, to compute a percentage of lower or equal power nodes in the network.
- We compute the Chernoff Bound for *Y*, using Equation (3).

We then plot a point representing the two values. We repeat this process for all nodes in all 1000 networks, resulting in 300,000 points, as plotted in Figure 2.

Figure 2 also shows the average count percentage found at each probabilistic value. We rounded the calculated probabilities to the closest second decimal digit, and consolidated similar points, then averaged the points and plotted them.

It can be seen from Figure 2 that the theoretical upper bound is a true upper bound. Meaning each computed probability is always higher or equal to the counted percentage. It can also be noticed that, at least up to a probabilistic value of 0.9, the counted values are half the theoretical upper bound. When plotting averages in Figure 2, we find that the averages are below half the theoretical bound for all cases. We speculate that a reason for this could be that almost half the nodes in the network are at a power level above E(X) with the other half being below. This division is due to *p*, the probability of discharge, being 0.5 as a default value selected for this plot and our network parameters Table 2.

Next, we will address the results obtained by our algorithmic approaches that utilize this plotted upper bound equation (Equation (3)).

### 10.2. Algorithm Results

We will show the performance of our algorithms on more than one hundred thousand networks with no knowledge, and how our algorithms compare to a full knowledge algorithm. Using the parameters in Table 2, we generate a simulated WRSN with different sizes. Then, we run our algorithms on each network created and record the result returned by each algorithm. We report our results as a percentage of the increase obtained when running the optimal full knowledge algorithm on the same network. When presenting performance, we use box plots, where the red line represents the median of the performance values and the blue dotted line represents the mean of the values.

**Figure 3:**
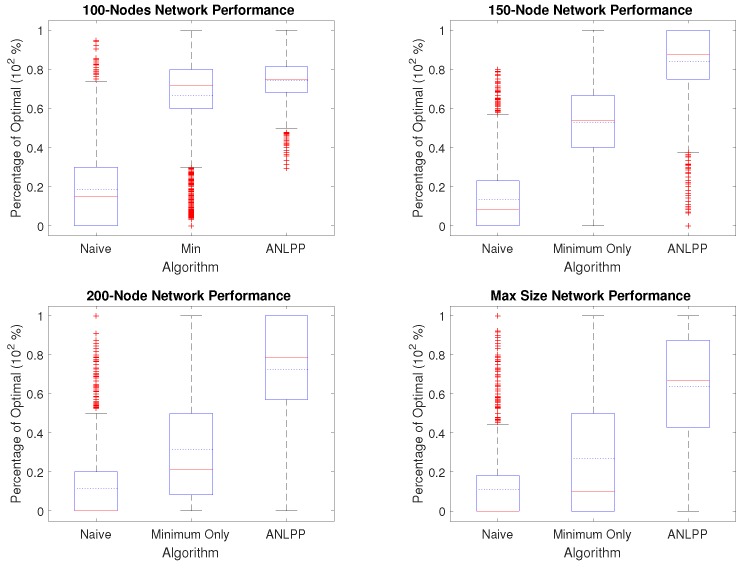
The performance of each algorithm over ten thousand networks of fixed size. The upper left image shows the results for a 100-node network, upper right 150-node, lower left 200-node, lower right maximum size to charge a single node (between 233 and 237).

Figure 3 shows the performance of all algorithms over four different network sizes, with ten thousand networks of each size. We pick four sizes: 100, 150, 200 and a maximum network size such that a single node can be charged if the network is fully explored. If we consider, for example, the case of a 150-node network, Naive algorithm’s median performance is 8.2% of optimal and it has a mean performance of 13.4%, while Minimum Only median performance is 53.8% and a mean performance of 53%, and ANLPP’s median performance is 87.5% and has a mean performance of 84%.

It can be seen from Figure 3 that ANLPP not only outperforms the other two implementations, but also has a narrower variance and also a median performance higher than its mean performance. Figure 3 shows how the Naive algorithm can only perform up to a smaller percentage of optimal because it uses all its power charging the first nodes it encounters. On the other hand, the Minimum Only algorithm does better, by charging nodes with minimum power. On the other hand, it also includes non-minimum nodes, which leads to wasted power, thereby causing a smaller life-increase in the WRSN. While Naive has no algorithm parameters, both Minimum Only algorithm and ANLPP used the threshold parameters given in Table 2.

While ANLPP most of the time outperforms the other two algorithms, we wish to observe its exact behaviour to identify when and where it performs well and poorly. To understand how ANLPP behaves, we need to identify the types of nodes in a WRSN, from a charging algorithm perspective. ANLPP divides nodes into two categories. Based on the action it takes regarding a node, ANLPP either adds the node to the charging list υ, or not. Each node in the network belongs to one of three different sets:

- Υ, the node should be charged for optimal performance,
- *k* low power nodes, dropped for optimal performance,
- high power nodes, need not be charged.

**Figure 4:**
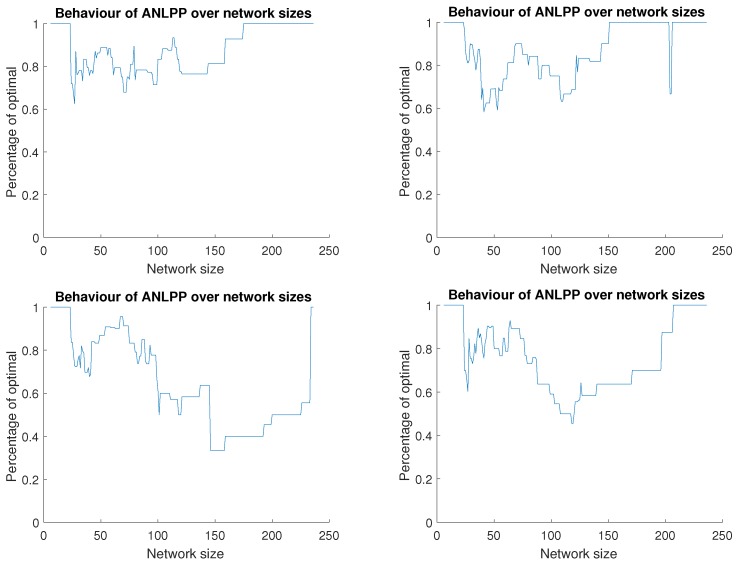
All Network Least Possible Probability (ANLPP) behaviour on four random networks with the same parameters, ANLPP tuned for maximum size. All cases starting and ending with optimal life increase for the maximum size of the network, while having different behaviours in between.

Given the above descriptions, Figure 4 shows the charging performance of ANLPP on four networks with the same parameters. Each sub-figure shows the trend in performance as the network size increases. In all cases, the algorithm starts at optimal performance, since the network size is small enough to fully explore. While they all also show a final optimal performance, there is a lot of variation in each network and between different networks. As the network size increases, the performance varies and demonstrates the discrete nature of the charging problem, and how small changes in network size may have a significant and varying impact on the network lifetime increase. We will attempt to analyze this behaviour.

When the network size increases, three possible outcomes could occur:

- No change in performance, seen as horizontal short lines of consistent performance in Figure 4. This is due to two possible cases: (1) the new node is part of Υ and ANLPP added it to υ as well. (2) the new node is a high power node that does not need to be charged and ANLPP does not visit it or ignores it despite visiting.
- Decrease in performance, noticed as a sharp or staircase decaying performance in Figure 4. This is due to three possible cases: (1) the new node is in Υ, but ANLPP does not reach it, or reaches it and decides not to add it to υ (due to threshold); (2) the new node is a high power node that does not need to be charged, while ANLPP adds it to υ; (3) the new node belongs to the *k* nodes that may be dropped, but ANLPP adds it to υ.
- Increase in performance, observed as a sharp or staircase increases in Figure 4. This is due to two possible cases: (1) the new node replacing a node in Υ, and the replaced node was not in υ but the new one is, bringing υ closer to Υ; (2) the new node belongs to the *k* nodes that may be dropped, and the addition of this node to the *k* nodes modifies Υ, bringing it closer to υ.

Next, we will look into the impact of network parameters on the performance of ANLPP.

### 10.3. Impact of the Algorithm Tuning Parameters

**Figure 5:**
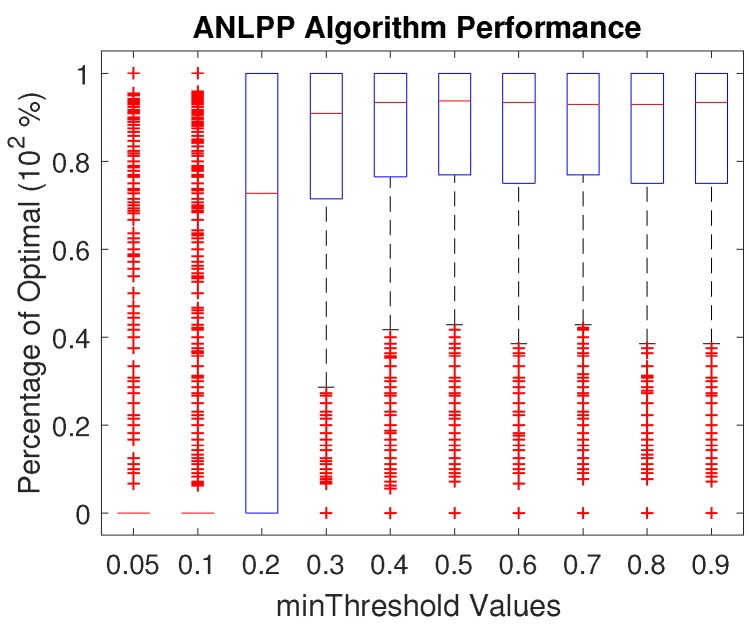
The impact of minThreshold on the performance of All Network Least Possible Probability (ANLPP).

**Figure 6:**
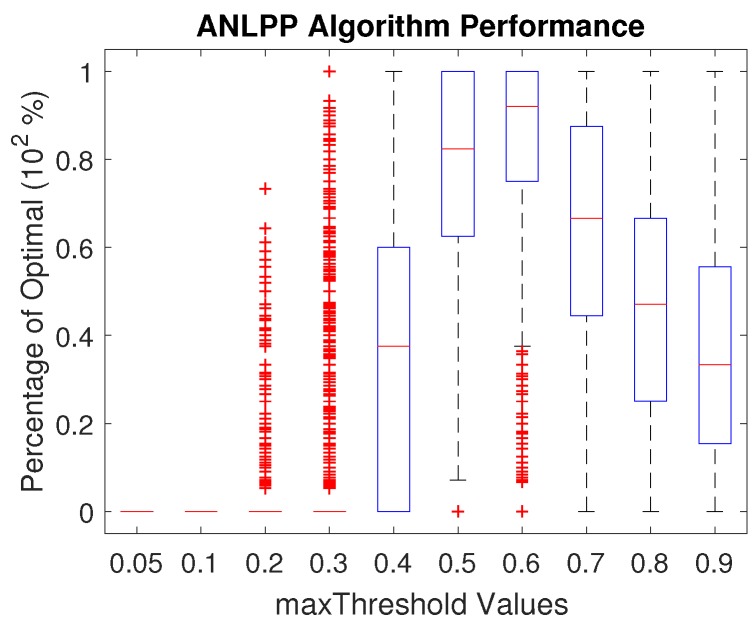
The impact of maxThreshold on the performance of All Network Least Possible Probability (ANLPP).

In this section, we show the results of changing the algorithm tuning parameters. In the ANLPP algorithm, we used two parameters, which are probability limit values. The minThreshold parameter makes sure the lowest node to charge is within a certain range. The maxThreshold parameter makes sure the highest node in the charging list is within a certain range. Figure 5 shows the impact of minThreshold, and Figure 6 shows the impact of maxThreshold.

Figure 5 shows that, for the best performance of ANLPP, the minThreshold needs to reach a sufficiently high value. Increasing minThreshold causes ANLPP to reach a peak performance and then stabilize. Low values of minThreshold prevent the algorithm from selecting nodes to charge in favour of finding very low power nodes. This leads to either selecting nodes that actually could be dropped, or exhausting most of the MCR power while searching. This in turn reduces the performance of the algorithm, while further increase in minThreshold has a smaller impact, due to maxThreshold becoming the driving factor when minThreshold is too high.

Figure 6 shows that maxThreshold has an optimal value for best possible performance of ANLPP, where an increase or decrease leads to degraded performance. A low value of maxThreshold leads to low performance for the same reasons a low value of minThreshold does. A high threshold value also leads to decreasing the algorithm performance. This is due to the algorithm terminating sooner than it should, after finding nodes with high probability of existence, leading to less exploration. In addition, a high threshold causes the algorithm to not pick low power nodes; hence, it is not able to extend the life of the network to high levels.

**Figure 7:**
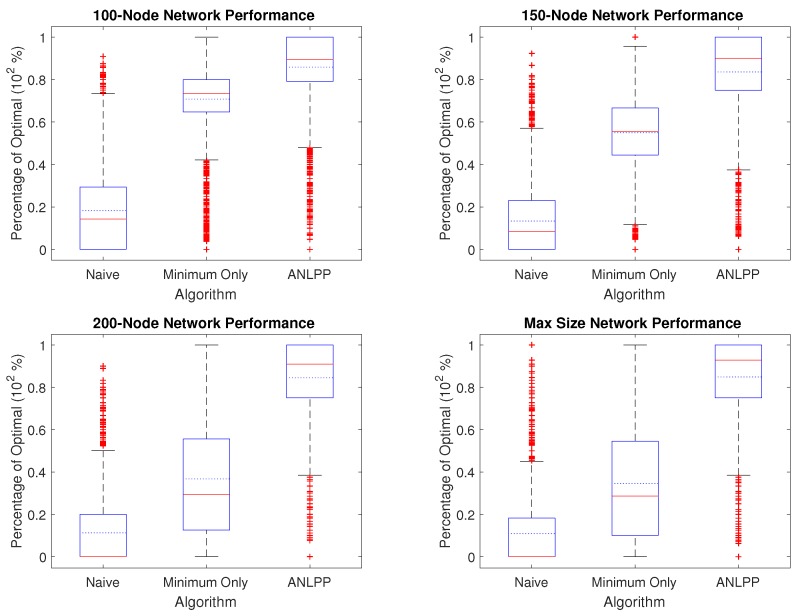
All algorithms’ performance over 10,000 networks of fixed size. Upper left is for size 100 nodes, upper right 150 nodes, lower left 200 nodes, and lower right maximum size to charge a single node (between 233 and 237).

Figure 7 shows the performance of both Minimum Only and ANLPP after having minThreshold and maxThreshold tuned for each network size. We tune both minThreshold and maxThreshold numerically offline for both algorithms. The offline simulations used similar network parameters as the networks used to evaluate the algorithms. After tuning Minimum Only, we notice increases in mean, median and a shrinking variance, and Table 3 summarizes the performance improvements for each network size. The improvements in the mean performance are almost always positive with an increase varying between +2% to +21%. Variance shows a fluctuation between narrowing and widening, or even no change. It can be seen that tuning improves at least two performance indicators of the algorithm, leading to either higher overall performance by increases in mean or better performance predictability with decreases in variance. While tuning improves the performance of Minimum Only, we notice an even higher improvement when tuning ANLPP. Even though the algorithm parameters are static, this still shows the algorithm’s flexibility to deal with variations in network parameters.

### 10.4. Impact of the Network Parameters

For all simulated WSN created, we used the parameters in Table 2. We now show the impact of these parameters on the performance of ANLPP.

We explored the impact of varying d,k, localization time,r,W, $E_{c},\;p,$ and *T* on the performance of ANLPP. Some parameters show little to no effect on the algorithm performance. Changes in *W* and *r* almost have no impact on the algorithm performance. This is because these parameters only impact the value of E(X), and not the algorithm or its behaviour. Other parameters show varying impact on performance. We notice a range of parameter changes that leads to very little or no impact on performance. Table 4 shows the possible range of parameter change that maintains performance when tuned for parameter values. We focus on some parameters and omit others that act similarly; we focus on d,k, and localization time.

**Figure 8:**
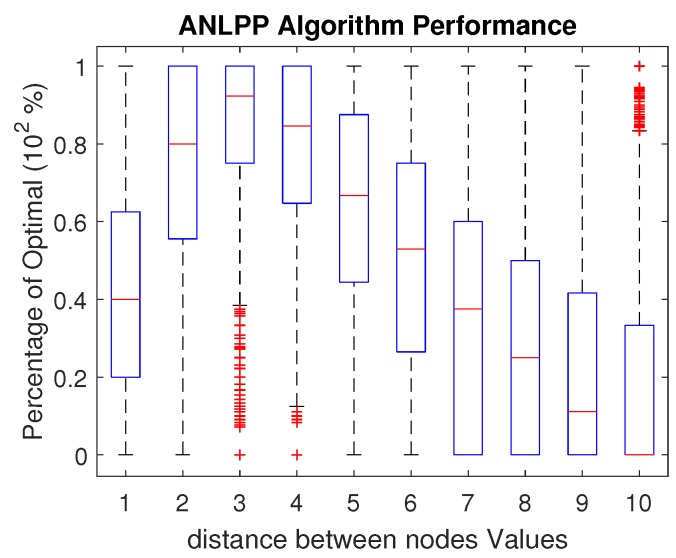
The impact of *d*, the distance between nodes, on ANLPP performance, when tuned for d=3.

**Figure 9:**
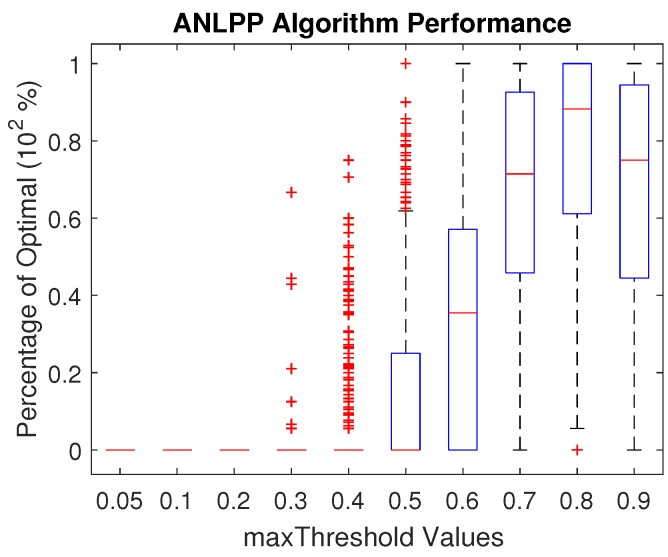
The performance of ANLPP when d=7 and a different values of maxThreshold used for each run.

The distance between the sensor nodes, *d*, impacts the performance of ANLPP. Figure 8 shows the impact of *d* on ANLPP, when it is tuned for d=3. It is clear that the algorithm peaks at d=3, and the algorithm’s performance degrades with the change in *d*. However, with a small range of change (±0.5), the algorithm performance almost stays the same. While *d* has the narrowest range of values, ANLPP may tolerate and still provide high performance, and it can still be tuned to work with a different value of *d* all together. Tuning ANLPP is done by setting the proper values of minThreshold and maxThreshold. Figure 9 shows the choice of a different value d=7 and the impact of maxThreshold on performance. This tuning process is currently performed offline and assumes the parameter *d* does not change during execution. This assumption is reasonable for most of our network parameters, since none of the parameters are of dynamic nature in a given WRSN.

**Figure 10:**
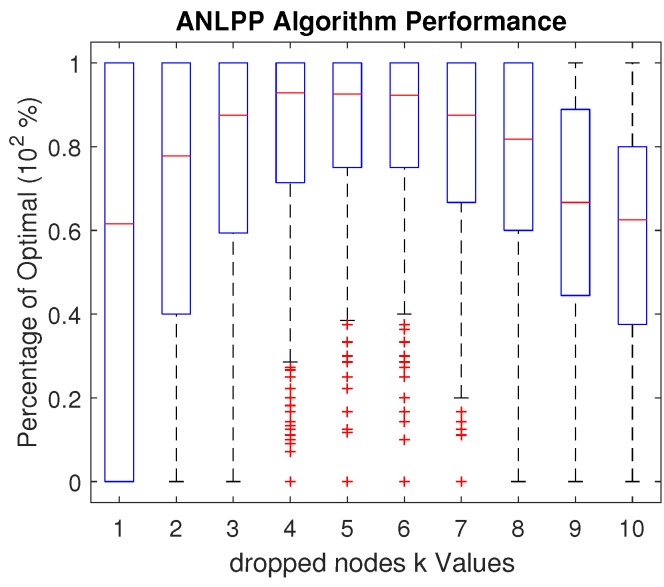
The impact of *k*, the number of drop nodes, on ANLPP performance, when tuned for k=5.

Another network parameter that ANLPP is less sensitive to with a slightly higher range is *k*, and the number of nodes that may be dropped. While *k* is a constant when it comes to a given WRSN, we can show that ANLPP can tolerate a slight change in this value. This tolerance may enable the algorithm to deal with different settings without the need for reconfiguration, or enable the algorithm to still be utilized during transition phases of a WRSN. Figure 10 shows the impact of changes in *k* on ANLPP tuned for k=5 on a WRSN. While a big increase or decrease of *k* leads to a degradation of ANLPP performance, a slight change leads to almost no impact. While ANLPP in Figure 10 is tuned for dropping five nodes, it performs at the same level when dropping four or six nodes.

Figure 10 also shows the inability of ANLPP to maintain performance with the decrease in *k*. This is because decreasing *k* forces the need to find the exact minimum nodes, leading to a higher cost for missing a low power node. Given that the algorithm is tuned to accept nodes with a certain power, changing the level of acceptance requires a change in acceptance threshold. An increase in *k* will cause ANLPP to charge nodes that could be dropped. Retuning the algorithm parameters enables the ANLPP to pick nodes properly again. A slight change in *k* may actually have no impact on the algorithm. This insensitivity to slight changes in *k* is very advantageous, since, in a real world WRSN, *k* will not change significantly.

**Figure 11:**
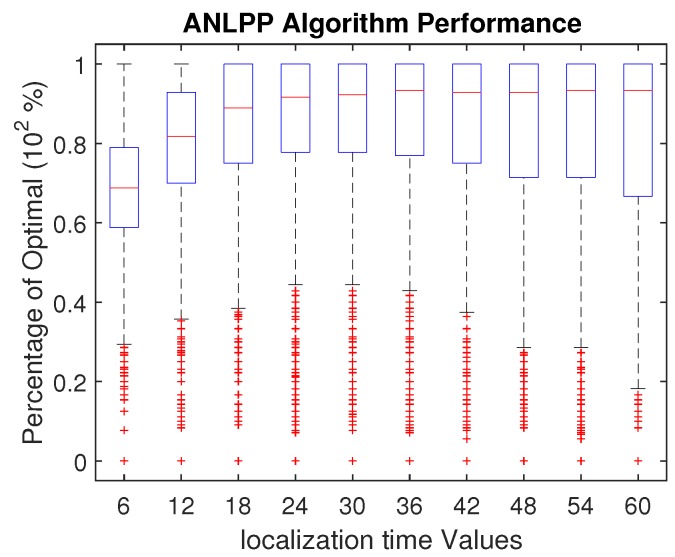
The impact of localization time on ANLPP performance, when tuned for localization time =25.

The next parameter we will investigate is localization time, the time it takes the MCR to concentrically localize with the node. Figure 11 shows ANLPP tuned for a localization time of 36 s. While a decrease in localization time has a higher impact than an increase, there is still a wide range where the performance is maintained. Even though the algorithm was tuned for 36 s, performance is almost not impacted down to 18 s. The decrease in localization time leads to a decrease in the cost of charging a node, which in turn increases the number of nodes to charge in the optimal full knowledge algorithm. Due to limiting the power level of the nodes to charge, ANLPP can deal with slight changes in charging cost, but not large decrease in charging cost. On the other hand, an increase in localization time leads to an increase in charging cost. This increase in cost in turn reduces the number of nodes the optimal algorithm can charge. While this also impacts ANLPP, the constant decrease in both algorithms enables ANLPP to keep up with the optimal algorithm’s performance.

This ability of ANLPP to deal with a large range of localization time renders the algorithm very practical. In the real world, localization time cannot be guaranteed to be fixed. Due to noise and natural environment factors, localization time may slightly vary. ANLPP shows it can deal with these slight variations and maintain high performance. While a large decrease in charging cost leads to a decrease in performance, retuning ANLPP will enable it to accommodate this change. By selecting a higher value for maxThreshold, ANLPP will accept higher power sensor nodes in its charging list υ. Accepting higher power sensor nodes enables ANLPP to increase the number of nodes to charge, bringing it close to the optimal charging list.

## 11. Conclusions

In this work, we presented ANLPP (All Network Least Possible Probability), a novel algorithm to charge a WRSN without a priori knowledge using a single MCR (Mobile Charging Robot). By disjointing the charing problem from the path planning problem, the charging problem is no longer NP-Hard. This reduction in complexity and no prior knowledge of power levels enable the scalability of the charging algorithm. The proposed algorithm is tunable and agnostic to the underlaying WRSN protocols. Through simulations, we showed that ANLPP on average, and with a low variance, extends a WRSN life by up to 90% of what an optimal full knowledge algorithm can achieve. Our analyses and simulation results reveal the existence of a range for each network parameter that has little to no impact on our algorithm’s performance. On the other hand, we report how ANLPP can accommodate, in a single run, larger value-changes for some parameters. For example, large changes in localization time and discharge rate have almost no impact on performance, while, for distance, it can accommodate a smaller change. The proposed approach is flexible to operate under different network parameters and can be applied to large-scale WRSN using a single MCR.

In the future, our proposed algorithm could be extended to incorporate a richer set of sensor nodes’ power consumption models, while keeping it agnostic to the WRSN underlying protocols. We also plan to explore the potential benefit of online tuning with respect to several parameters changing simultaneously. We wish to evaluate the performance of ANLPP on a physical UAV based charging system. In another direction, we would like to investigate integrating ANLPP with the path planning process of an MCR, and then comparing it to other charging algorithms. We wish to extend ANLPP as a distributed algorithm using multiple MCRs to charge a single WRSN. We are also interested in leveraging a data prediction algorithm like [53] for our nodes power consumption model and analyzing its impact on the performance of ANLPP.

## Figures and Tables

**Table:** 

	
	

**Table:** 

	
	

**Table:** 

	
	

**Table 1:** Notations used in problem formulation.

Notation	Meaning
$B_{S}$	base charging station, MCR recharge & start point
*N*	set of all sensor nodes
$n_{i}$	sensor node at location *i*
$N_{e}$	set of all residual energy levels
$e_{i}$	residual energy of sensor node *i*
$N_{d}$	set of distances between nodes
$d_{i}$	distance from sensor node i−1 to sensor node *i*
*u*	number of sensor nodes to charge
λ	cost of concentrically localizing MCR with a node
Υ	optimal set of nodes to charge
Ψ	optimal WRSN time increase
Γ	optimal exploration termination point
υ	set of nodes to charge with no knowledge
ψ	WRSN time increase with no knowledge
γ	exploration termination point with no knowledge
*l*	target power of a node to charge
$E_{S}$	battery capacity of a sensor node
$E_{C}$	battery capacity of a MCR
*C*	total power consumed for charging
*M*	total power consumed for moving
$\Lambda_{m}$	the MCR moving power consumption per move unit
*r*	sensor node discharge rate
*p*	sensor node discharge probability at each time unit
g(n)	node discharge function
*k*	number of sensor nodes that may reach zero power level
*Y*	power level of the currently explored sensor node

**Table 2:** Network configuration parameters.

Parameter	Value
$E_{C}$	25 WH
*s*	236 nodes
*T*	$E_{C}$ ticks
Travel Power	121.91 W
*d*	Travel Power × 3 s
Hover Power	92.28 W
Localization Time	36 s
λ	92.28 W × 36 s
*r*	1.625 mWH
*W*	2.34 WH
*p*	0.5
minThreshold used in Minimum Only	0.17
minthreshold used in ANLPP	0.37
maxthreshold	0.7
*k*	5

**Table 3:** Tuning impact on algorithms’ performance change.

Algorithm	Network Size	Mean	Median	Variance
Minimum Only	100	+0.05	+0.01	−0.05
Minimum Only	150	+0.02	+0.015	−0.05
Minimum Only	200	+0.052	+0.085	+0.015
Minimum Only	Max	+0.076	+0.186	−0.055
ANLPP	100	+0.115	+0.145	+0.085
ANLPP	150	−0.005	+0.025	0
ANLPP	200	+0.118	+0.123	−0.178
ANLPP	Max	+0.213	+0.26	−0.196

**Table 4:** ANLPP network parameter range tolerance.

Parameter	Optimal Performance Tuned Value	Maintained Performance Start	Maintained Performance End
*d*	3 $\times \Lambda_{m}$	2.4	3.7
*k*	5 nodes	3	7
localization time	36 s	18	54
*r*	1.625 mWH	50 μWH	4 mWH
*W*	2.34 WH	1.17 WH	9.36 H
$E_{C}$	25 WH	19	37
*p*	0.5	0.43	0.63
*T*	$1\times E_{C}$	$0.9\times E_{C}$	$1.1\times E_{C}$

## Abbreviations

The following abbreviations are used in this manuscript:

WRSN	Wireless Rechargeable Sensor Networks
WSN	Wireless Sensor Networks
UAV	Unmanned Aerial Vehicle
MCR	Mobile Charging Robot
NPC	Nondeterministic Polynomial Complete
NP-Hard	Nondeterministic Polynomial Hard
ANLPP	All Network Least Possible Probability
RF	Radio Frequency
CC	Centralized Charging
DC	Distributed Charging
USDA	United States Department of Agriculture
NIFA	National Institute of Food and Agriculture
NSF	National Science Foundation
